# Supplementary material for: Predation and fragmentation portrayed in the statistical structure of prey time series
Source: BMC Ecol. 2009 May 6;9:10. doi: 10.1186/1472-6785-9-10 (PMC2689204; doi:10.1186/1472-6785-9-10)
Supplement: Additional file 2 — Voles and related classes ODDox Documentation. ODDox documentation of the agent-based model (ALMaSS) applied by Hendrichsen et al. The documentation is started by activating main.html. [file 1472-6785-9-10-S2.zip › Vole_ODDox/functions_func.html]

ALMaSS ODDox: Class Members - Functions

- Main Page
- Related Pages
- Classes
- Files

- Alphabetical List
- Class List
- Class Hierarchy
- Class Members

- All
- Functions
- Variables

- a
- b
- c
- d
- e
- f
- g
- h
- i
- k
- l
- m
- n
- o
- p
- r
- s
- t
- u
- v
- w
- ~

### - a -

- AddField()
  : Farm- AddNewEvent()
    : Farm- AddToGeneticImpacted()
      : Vole\_Population\_Manager- AddToImpacted()
        : Vole\_Population\_Manager- AddToJuvs()
          : Vole\_Population\_Manager- AddToNotImpacted()
            : Vole\_Population\_Manager- AddToYoung()
              : Vole\_Population\_Manager- AgroChemIndustryCerealFarm1()
                : AgroChemIndustryCerealFarm1- AgroChemIndustryCerealFarm2()
                  : AgroChemIndustryCerealFarm2- AgroChemIndustryCerealFarm3()
                    : AgroChemIndustryCerealFarm3- AlleleFreq()
                      : AlleleFreq- AppendToFile()
                        : probe\_data- AssessHabitat()
                          : Vole\_Base- AutumnHarrow()
                            : Farm- AutumnPlough()
                              : Farm- AutumnRoll()
                                : Farm- AutumnSow()
                                  : Farm

---

Generated on Thu Jan 22 14:13:45 2009 for ALMaSS ODDox by 
 1.5.6
